# Acetabular cup anteversion measurement in total hip arthroplasty: reliability of metal artifact reduction MRI compared with CT

**DOI:** 10.1007/s00256-026-05161-y

**Published:** 2026-02-12

**Authors:** Frederik Abel, Patrick O. Zingg, Reto Sutter, Florian Schmaranzer

**Affiliations:** 1 Department of Radiology, Faculty of Medicine, Balgrist University Hospital, University of Zurich, Forchstrasse 340, 8008 Zurich, Switzerland; 2 Department of Orthopedic Surgery, Faculty of Medicine, Balgrist University Hospital, University of Zurich, Forchstrasse 340, 8008 Zurich, Switzerland; 3 Department of Diagnostic, Interventional- and Paediatric Radiology, Inselspital, University of Bern, Rosenbühlgasse 27, 3010 Bern, Switzerland

**Keywords:** Acetabular anteversion measurement, Total hip arthroplasty, Prosthetic hip imaging, Computed tomography, Magnetic resonance imaging, Metal artifact reduction sequences

## Abstract

**Objective:**

The acetabular cup version in patients with total hip arthroplasty (THA) is a key parameter influencing hip stability and functional outcomes. Although CT remains the reference standard for assessing cup orientation, MRI with metal artifact reduction techniques is increasingly used for evaluating postoperative pain. This study compared MRI- and CT-based measurements of acetabular cup version regarding agreement, reproducibility, and reliability.

**Material and methods:**

Patients who underwent THA between 2015 and 2025 with postoperative CT and MRI were retrospectively analyzed. MRI was performed using optimized metal artifact reduction sequences. Acetabular cup version was measured on axial high-bandwidth T1-weighted turbo spin-echo sequences and on corresponding CT scans by two musculoskeletal radiologists. Inter- and intra-reader, as well as inter-modality agreement, were assessed using intraclass correlation coefficients (ICC). Bland-Altman plots evaluated systematic bias.

**Results:**

Thirty patients were included (mean age, 64.1 years; 14 women). Mean acetabular version was 31.2° (standard deviation (SD), 9.4–10.3) for CT and 30.1–30.2° (SD, 8.9–9.3) for MRI for both readers. Inter-reader agreement was almost perfect for CT (ICC, 0.96) and substantial for MRI (ICC, 0.76). Intra-reader agreement was almost perfect for both modalities (ICC, CT, 0.99; MRI, 0.94). Bland-Altman analysis showed no statistically significant differences between CT and MRI measurements with a slight positive bias for MRI (mean pooled difference, 1.1°; *p* = 0.058; limits of agreement, −4.7 to 6.8°) and almost perfect agreement for both readers (ICC, 0.86–0.90).

**Conclusion:**

MRI measures acetabular cup version with excellent reliability and close agreement with CT, reinforcing MRI’s role in postoperative THA evaluation.

## Introduction

Total hip arthroplasty (THA) is highly effective for end-stage osteoarthritis, restoring function and relieving pain in most patients, with procedure volumes projected to rise by 176% by 2040 due to an aging population with increasing demand for improved mobility and quality of life [1]. However, persistent or new symptoms occur in a subset of patients [2], with short- and long-term outcomes depending on accurate prosthetic component positioning [3].

Acetabular cup version and femoral shaft torsion are key determinants of hip stability and play a central role in minimizing postoperative complications and revision surgery [4]. Although the ideal acetabular cup orientation is increasingly considered patient-specific and specific to the surgical approach, a safe range of 15° ± 10° for acetabular anteversion and 25–50° for combined anteversion (cup + stem) is generally accepted [5, 6]. Suboptimal acetabular positioning, including both excessive and insufficient anteversion, has been associated with higher dislocation rates, accelerated wear, restricted range of motion, and impingement-related complications [7–10].

CT is the most accurate modality for assessing acetabular cup version, offering far greater precision than radiographic measurements [11]. However, MRI is increasingly utilized for evaluation of symptomatic THA following radiographs, as recommended by the American College of Radiology Appropriateness Criteria® [12]. Advances in metal artifact reduction techniques, combined with MRI’s inherently superior soft tissue contrast, provide high sensitivity to many postoperative complications such as periprosthetic infection or tendon pathology [13]. Despite these advances, to our knowledge, the feasibility of MRI for measuring acetabular cup version has not been systematically investigated. MRI with metal artifact reduction sequences may therefore allow simultaneous assessment of cup version, supporting the detection of acetabular cup malposition in the context of a symptomatic THA.

This study aimed to evaluate the agreement, reproducibility, and reliability of MRI-based acetabular cup version measurements compared with CT in patients with THA.

## Materials and methods

### Patient selection

This single-center, retrospective study was approved by the local ethics committee (Cantonal Ethics Committee Zurich). All procedures complied with the ethical standards of the institutional and/or national research committee and were conducted in accordance with the principles of the 1964 Declaration of Helsinki and its later amendments. All included patients have given written informed consent that allows their health-related data to be used for research purposes.

To identify patients for this study, a database query of the hospital’s information system was performed to retrieve records of all THAs performed between 2015 and 2025. Patients were included if they underwent THA due to primary or secondary osteoarthritis, neoplasia, or had a history of THA revision due to aseptic loosening or periprosthetic infection. From this cohort, those who received both postoperative CT examinations and small field-of-view (FOV) MRI of the hip were identified from the local Picture Archiving and Communication System (Merlin, Phoenix PACS). Patients were excluded if THA revision was performed in the interval between CT or MRI, or if only large-FOV MRI of the entire pelvis was available. This exclusion ensured a homogeneous imaging protocol and avoided compromises in spatial resolution that could impair acetabular cup measurements due to reduced edge visibility associated with large-FOV acquisitions.

Imaging was performed for evaluation of THA complications and assessment of prosthetic positioning. The prostheses consisted of titanium alloy-based acetabular cups and stems combined with ceramic or cobalt-chromium heads. In patients with a history of revision THA, additional acetabular screw fixation or cement augmentation was present in some cases.

### Image analysis

All patients underwent a standardized imaging protocol with optimized metal artifact reduction sequences for hip prostheses acquired at 1.5 Tesla (T) (Magnetom AvantoFit, or Magnetom Sola; Siemens Healthineers) using an 18-channel body coil and a 32-channel spine coil (Table 1). The complete clinical protocol included a compressed sensing-based coronal short-tau inversion recovery (STIR) sequence with slice encoding for metal artifact correction (SEMAC) and an axial two-dimensional (2D) STIR turbo spin-echo (TSE) sequence with optimized inversion pulse (WARP) covering the entire prosthesis. In addition, smaller FOV 2D TSE sequences were obtained, covering the hip joint and the abductor muscles: coronal T2-weighted and axial and sagittal T1-weighted sequences with high bandwidth. The protocol also included a rapid 2D, axial T2-weighted sequence (HASTE) of the entire pelvis and femoral condyles [14].

**Table 1:** 1.5 T hip MRI protocol with optimized metal artifact reduction sequences

Parameter	Coronal STIR CS-SEMAC	Axial 2D STIR WARP^a^	Coronal 2D T2 TSE High-Bandwidth	Axial 2D T1 TSE High-Bandwidth	Sagittal 2D T1 TSE High-Bandwidth	Axial 2D T2 HASTE
Anatomical coverage	Prosthetic hip	Prosthetic hip	Prosthetic hip	Prosthetic hip	Prosthetic hip	Full pelvis
TR/TE (msec)	4220/36	4000/31	4000/58	600/8.6	550/7.3	663/7.4
Flip angle (°)	140	150	150	180	180	145
ETL	9	11	15	3	3	3
NSA	1	3	2	2	2	1
RBW (Hz/pixel)	500	450	390	425	435	500
FOV (mm^2^)	280 × 280	180 × 126	220 × 121	200 × 160	200 × 200	400 × 280
Matrix size	256 × 205	384 × 269	512 × 282	512 × 410	320 × 320	384 × 269
Section thickness (mm)	3.5	7	4	6	4	5
Scan time (min:sec)	5:30	4:00	2:30	2:20	2:00	1:00

*2D* two-dimensional, *CS* compressed sensing, *ETL* echo train length, *FOV* field of view, *HASTE* half-fourier acquisition single-shot turbo spin echo, *RBW* receiver bandwidth, *SEMAC* slice encoding for metal artifact correction, *STIR* short-tau inversion recovery, *TE* echo time, *TR* repetition time, *TSE* turbo spin-echo

^a^WARP; Siemens Healthcare, Munich, Germany

CT scans of the pelvis were performed using different multi-slice CT scanners (Definition AS Plus or Edge Plus, Siemens Healthineers; Brilliance 64, Philips Healthcare) or a photon-counting CT system (Naeotom; Siemens Healthineers). All scans were acquired at 120–140 kVp/keV, with reconstructions at a slice thickness of 1–2 mm.

**Fig. 1:**
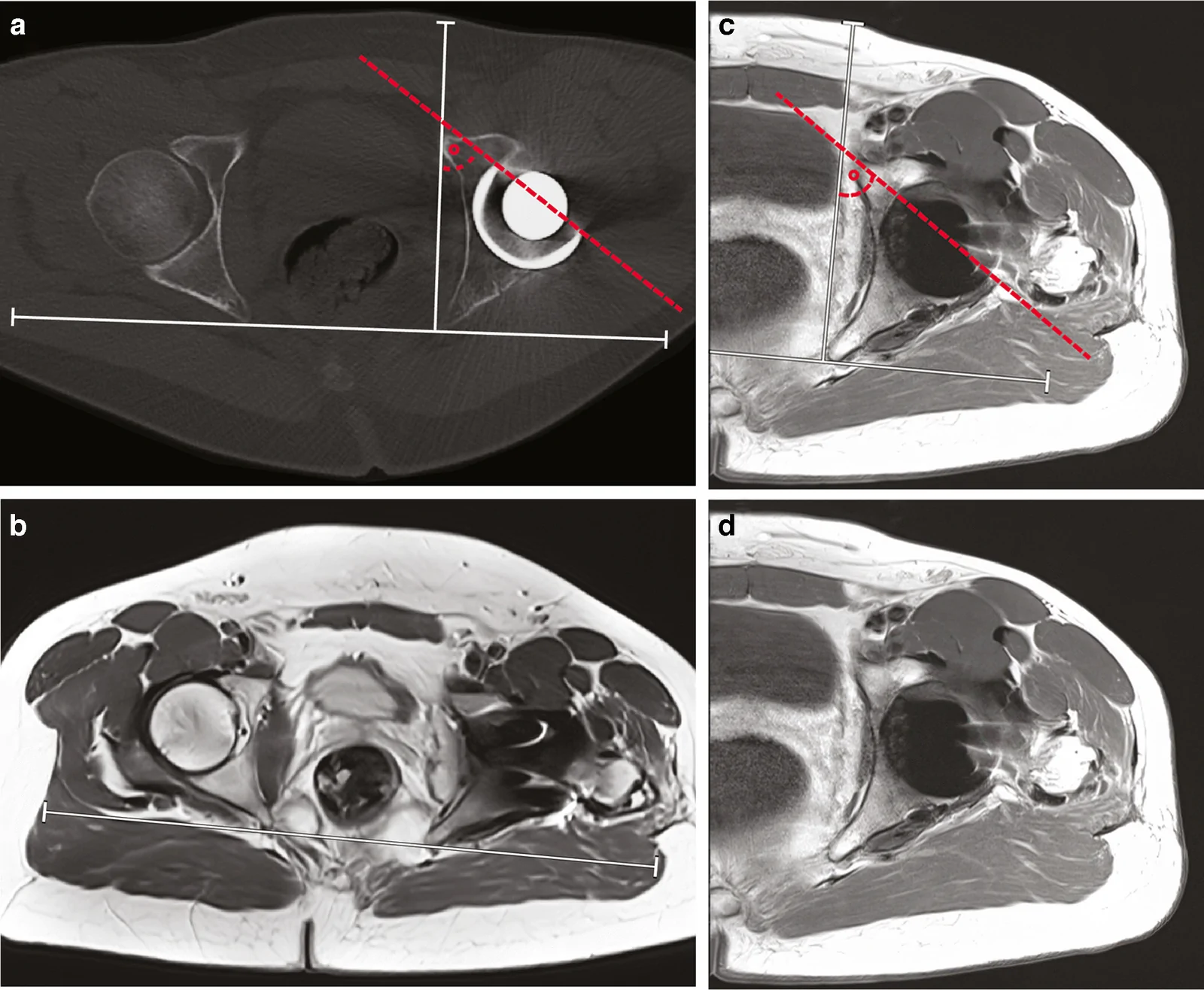
Measurement of the acetabular cup version angle in a patient with left total hip arthroplasty. On axial CT scans (**a**) (window center/width 2300/7000 HU, Br59 kernel), a transverse reference line was drawn through the ischial spines, and the angle was measured between the cup’s equator and a line perpendicular to the reference. On MRI, the reference line was placed on an axial T2-weighted HASTE sequence (**b**) and projected onto an axial high-bandwidth T1-weighted turbo spin-echo sequence (**c**), where the measurement was performed in the same manner. The corresponding unannotated MR image is shown in (**d**) for comparison. The acetabular anteversion measured 54° on both modalities. The interval between CT and MRI was 143 days

**Fig. 2:**
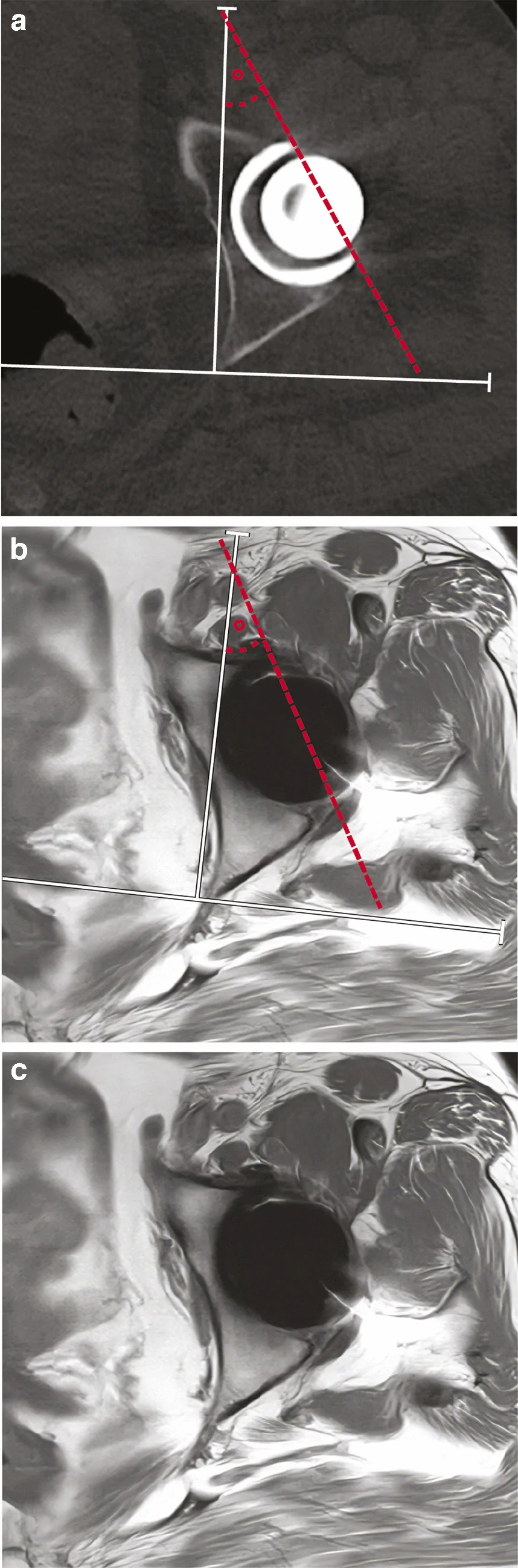
Assessment of the acetabular cup version angle in another patient with left total hip arthroplasty on an axial CT scan (**a**) (window center/width 1500/3500 HU, Br57 kernel), corresponding axial high-bandwidth T1-weighted turbo spin-echo sequence with annotation (**b**), and the same image without annotation (**c**). The acetabular anteversion measured 30° on both modalities. The interval between CT and MRI was 128 days

On MRI, the acetabular cup version angle was measured using two sequences: (1) the axial T2-weighted HASTE sequence with full FOV covering the entire pelvis and (2) the axial T1-weighted TSE (high-bandwidth) sequence with a smaller FOV centered on the hip prosthesis. First, a *transverse reference line* was drawn through the ischial spines on the axial T2-weighted HASTE sequence bilaterally. This reference line was then co-referenced to the corresponding axial T1-weighted TSE sequence (high-bandwidth) by copying and pasting it to matching coordinates in the PACS system. The *version angle* was defined as the angle between the cup’s widest opening (equator) and a line perpendicular to the reference line. Measurements on CT were performed in a similar manner (Figs. 1 and 2).

Acetabular version angle measurements were independently evaluated by two musculoskeletal fellowship-trained radiologists (F.A. and F.S., with 5 and 8 years of experience, respectively) in randomized order of the anonymized cases for both modalities. Inter-reader and inter-modality agreement were assessed. To evaluate intra-reader agreement, reader 1 repeated all measurements after a 1-month time period.

### Statistical analysis

An a priori sample size calculation for the intraclass correlation coefficient (ICC), assuming an expected ICC of 0.8, a null hypothesis ICC of 0.5, alpha = 0.05 (two-tailed), and 80% power, indicated a minimum required sample size of 28 patients [15].

Mean differences in acetabular cup version angles between MRI and CT measurements were compared using paired *t*-tests. Inter- and intra-reader agreement, as well as inter-modality agreement between MRI and CT, were assessed using ICC. A two-way random-effects model was applied for inter-reader and inter-modality agreement, and a two-way fixed-effects model for intra-reader agreement, both calculated using absolute agreement and a single-measurement ICC. Bland-Altman plots were generated to visualize inter-modality agreement between MRI and CT measurements for each reader, as well as pooled across both readers, to evaluate systematic bias and limits of agreement. Values 0.21–0.40, 0.41–0.60, 0.61–0.80, and > 0.80 were considered fair, moderate, substantial, or almost perfect agreement, respectively [16]. Analyses were performed using R (Version 4.0.3, the R Foundation for Statistical Computing, Vienna, Austria).

## Results

### Patient characteristics

**Fig. 3:**
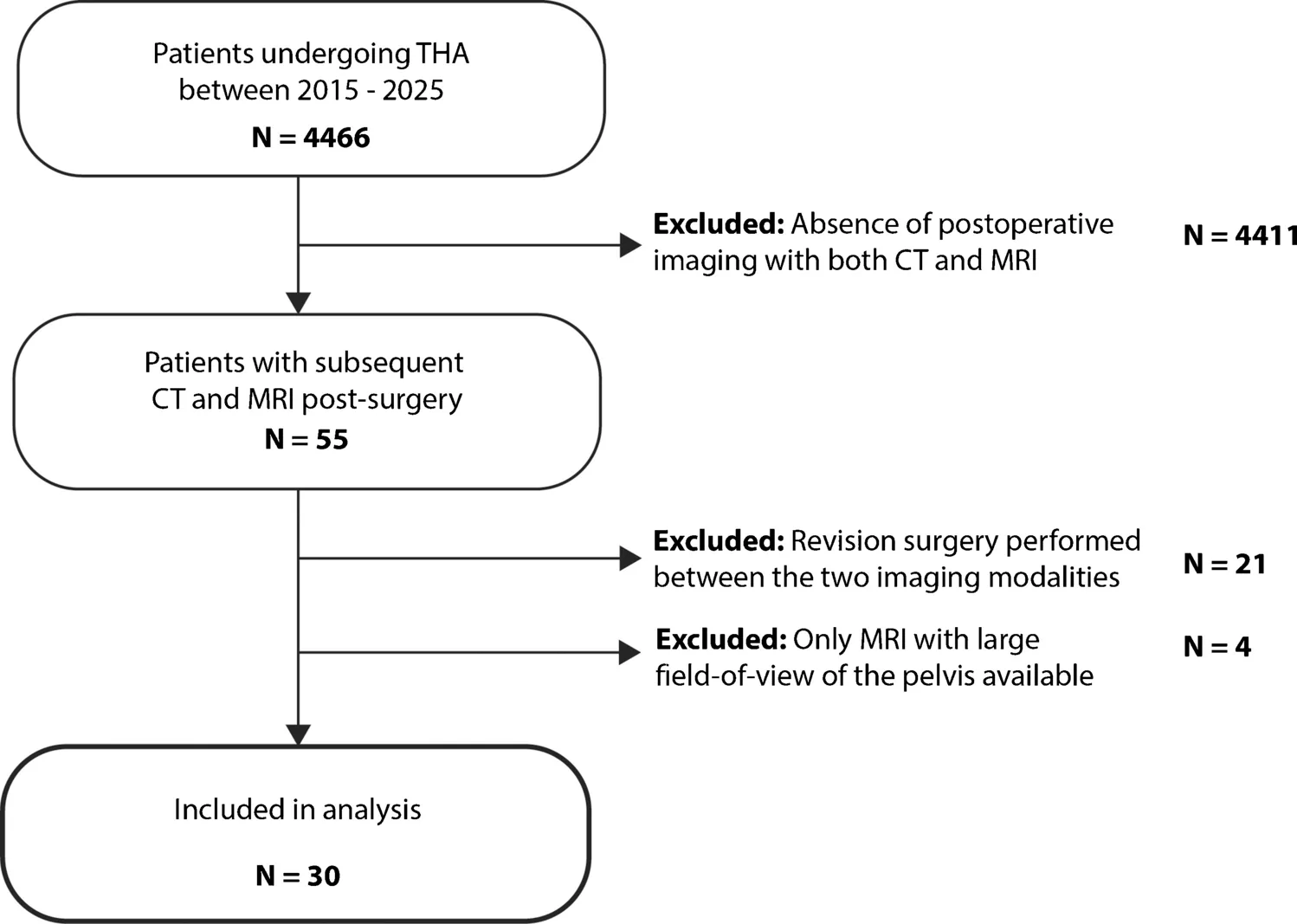
Flowchart illustrating the patient selection process. THA = total hip arthroplasty

The final study cohort consisted of 30 patients after application of the inclusion and exclusion criteria. The patient selection process is shown in Fig. 3.

All demographical and clinical data are summarized in Table 2. The mean age was 64.1 ± 16.2 years (standard deviation [SD]), and 14 patients were female. Six patients had a history of prior THA revision. The mean interval between surgery and the earliest postoperative imaging examination (MRI or CT) was 715 ± 1182 days, while the mean interval between the two imaging modalities was 205 ± 296 days.

**Table 2:** Patient demographic and clinical data

Characteristic	Value
Patients, total (*n*)	30
Sex (m/f), *n* (%)	16/14 (53/47)
Age (years), mean (SD)	64.1 (16.2)
BMI, mean (SD)	27.7 (5.2)
Diagnosis^a^, *n* (%)	
Osteoarthritis (degenerative)	14 (46.7)
Osteoarthritis (secondary)	10 (33.3)
Posttraumatic	4
Dysplasia	2
Avascular necrosis	3
Chondrocalcinosis	1
Periprosthetic infection	3 (10.0)
Aseptic loosening	2 (6.7)
Neoplasia	1 (3.3)

*BMI* body mass index, *SD* standard deviation

^a^Indication for total hip arthroplasty

### Acetabular version and inter-reader reliability

The mean acetabular version angle was 30.1° ± 8.9° and 31.2° ± 9.4° for MRI and CT for reader 1, respectively, and 30.2° ± 9.3° and 31.2° ± 10.3° for reader 2. No statistically significant differences were observed in mean version measurements between readers (Table 3).

**Table 3:** Mean acetabular version measurements by reader

Modality	Reader 1	Reader 2	Difference	*p*
MRI, mean (CI)	30.1° (26.7°, 33.4°)	30.2° (26.7°, 33.7°)	−0.2° (−2.6°, 2.2°)	0.90
CT, mean (CI)	31.2° (27.7°, 34.7°)	31.17° (27.3°, 35.0°)	0° (−1.0°, 1.1°)	0.95

*CI* 95% confidence intervals

Inter-reader agreement was substantial for MRI with ICC of 0.76 (95% confidence interval (CI), 0.55, 0.88) and almost perfect for CT with ICC of 0.96 (CI, 0.92, 0.98). Intra-reader agreement was almost perfect for both modalities, with slightly lower values for MRI (ICC, 0.94; CI, 0.89, 0.97) compared to CT (ICC, 0.99; CI, 0.97, 0.99).

### Inter-modality reliability and Bland-Altmann analysis

The inter-modality reliability was almost perfect for both readers with ICCs of 0.87 (CI, 0.75, 0.94) for reader 1 and 0.90 (CI, 0.81, 0.95) for reader 2.

**Fig. 4:**
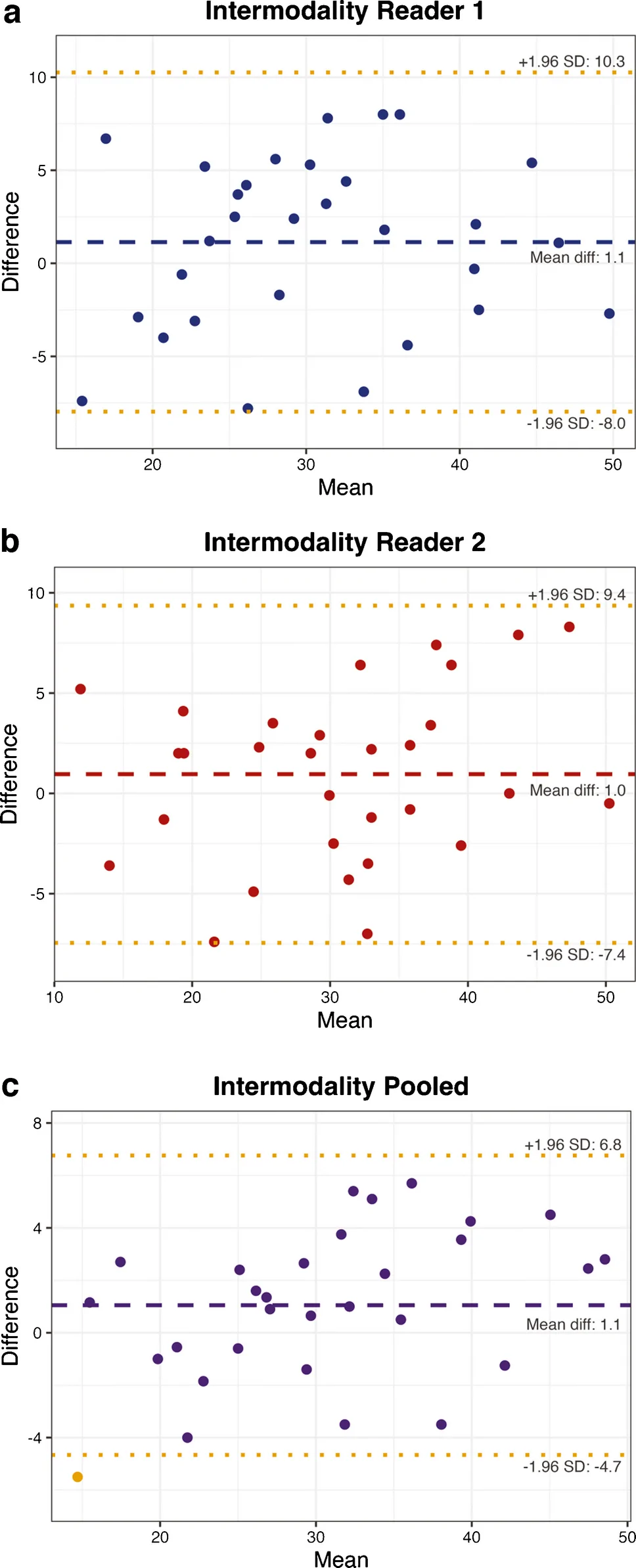
Bland-Altman plots comparing acetabular cup version measurements between MRI and CT for reader 1 (**a**), reader 2 (**b**), and pooled data across readers (**c**)

Bland-Altman analysis for reader 1 showed a slight positive bias of MRI compared with CT, with a mean difference of 1.1° (CI, −2.9°, 0.6°; *p* = 0.19) and limits of agreement from −8.0 to 10.3°, without evidence of a systematic bias across the range of measurements. For reader 2, the mean difference was 1.0° (CI, −2.6°, 0.6°; *p* = 0.23), with limits of agreement from −7.4 to 9.4°. In the pooled analysis across readers, the mean difference was 1.1° (CI, −0.04°, 2.1°; *p* = 0.058), with limits of agreement from −4.7 to 6.8° (Fig. 4).

## Discussion

This study evaluated the reliability of acetabular cup version angle measurements in MRI and CT in patients with THA, using an axial T1-weighted TSE sequence with high-bandwidth for metal artifact suppression. Inter-reader agreement was substantial for MRI, while intra-reader agreement was almost perfect. The Bland-Altman analysis revealed no significant mean difference between MRI- and CT-based measurements, with a slight positive bias for MRI (pooled mean bias of 1.1°), and almost perfect inter-modality reliability for both readers. Overall, these findings indicate that MRI provides reliable measurements of acetabular cup version in THA.

Similar to previous works [11, 17, 18], CT demonstrated high reliability for assessing acetabular cup version with almost perfect inter-reader agreement (ICC 0.99) in this study, whereas MRI expectedly achieved a lower but still substantial agreement (ICC 0.76) despite image distortion from metal artifacts. To date, MRI-based measurements of acetabular cup version in THA remain understudied, most likely due to the inherent challenges posed by susceptibility artifacts from metallic prosthesis components [19]. However, increasing incorporation of metal artifact reduction techniques and sequences now enables comprehensive postoperative MRI assessment of THA, with high sensitivity for detecting periprosthetic osteolysis, bone marrow changes, and periosteal edema [20, 21]. These include advanced methods such as 3D multiacquisition variable-resonance imaging combination (MAVRIC) or slice encoding for metal artifact correction (SEMAC) [22, 23], as well as more conventional strategies, such as using 1.5 T rather than 3.0 T field strength, fast/turbo spin-echo sequences instead of gradient-echo sequences, smaller voxel size, or higher receiver bandwidth [24]. In this study, an axial 2D T1-weighted TSE sequence with high bandwidth (425 Hz/pixel vs. ~ 200 Hz/pixel in standard non-metal protocols) was used to mitigate susceptibility artifact, as commonly implemented in THA imaging protocols [25]. MRI-based acetabular version measurements showed a mean pooled difference of 1.1° compared with CT, with limits of agreement from −4.7 to 6.8° and no systematic trend across measurements. The slightly higher values observed with MRI may reflect artifact accentuation at the edges of the acetabular equator, leading to an overall small positive bias. Given that a surgically targeted acetabular version range of ± 10° is generally considered clinically acceptable in THA implantation [26], the observed differences fall well within this range, indicating the feasibility of MRI for estimating cup version in a clinically relevant context.

Notably, the mean acetabular anteversion in the study cohort was 30–31°, which is considerably higher than the traditional safe zone of 15° ± 10°. This finding is likely explained by the predominantly performed direct anterior approach (*N* = 21), which is associated with higher anteversion compared with the lateral approach [27]. Another related and increasingly recognized factor for THA stability is the contemporary emphasis on combined anteversion, leading surgeons to intentionally place the cup with higher anteversion (cup + stem; target ~ 25–50°) when stem anteversion is low [6]. THA stability is determined by a complex interplay of the osseous configuration of the pelvis and proximal femur, involving not only acetabular version but also inclination, femoral torsion, and spinopelvic parameters. Normal spinopelvic mechanics change during standing-to-sitting transitions, with posterior pelvic tilt dynamically increasing functional anteversion [28]. In contrast, abnormal spinopelvic mobility—such as loss of posterior tilt in patients with spinal fusion—prevents this compensatory increase in functional acetabular anteversion, thereby raising the risk of anterior impingement and posterior dislocation [29]. In patients with stiff spinal imbalance, optimal THA positioning must therefore consider not only acetabular orientation but also anterior coverage and femoral stem position and its contribution to combined anteversion, in order to maximize hip stability and minimize impingement risk [30]. In this context, MRI with metal artifact reduction sequences may assist in detecting component malpositioning (e.g., excessive anteversion or retroversion) and thus should be performed with axial sequences of the pelvis and distal femoral condyles.

This study contains several limitations. First, its retrospective, single-center design may have introduced selection bias and limits generalizability. Second, femoral torsion was not evaluated because axial images of the femoral condyles were not acquired as part of the CT protocols, precluding assessment of combined anteversion. Third, the mean time interval between MRI and CT examinations was 205 days during which mild implant migration cannot be entirely excluded. Nevertheless, no radiographic or clinical evidence of relevant acetabular cup positional change was present in any patient, including two patients with aseptic loosening confined to the femoral stem. Fourth, CT scans were acquired on heterogeneous scanners, and a subset of patients (*N* = 4) underwent photon-counting CT, which provides higher resolution and artifact reduction compared with energy-integrating CT; however, no difference in inter-reader reliability was observed in a limited subanalysis. Finally, six patients had a history of THA revision with acetabular screw fixation or cement augmentation, which exacerbated susceptibility artifacts and may have lowered inter-reader agreement. Nevertheless, the inclusion of such cases reflected a more clinically realistic patient cohort.

In summary, this study shows that MRI with a high-bandwidth axial T1-weighted TSE sequence provides reliable measurements of acetabular cup version in THA, demonstrating almost perfect agreement with CT and only a minor positive bias within clinically acceptable limits. Although CT remains the gold standard, hip MRI may serve as a valuable tool for identifying clinically relevant malpositioning that can contribute to symptomatic or unstable THA.

## Data Availability

This study’s data is available by the corresponding author upon reasonable request.
